# Supplementary material for: Identification of photoperiod-induced specific miRNAs in the adrenal glands of Sunite sheep (Ovis aries)
Source: Front Vet Sci. 2022 Jul 22;9:888207. doi: 10.3389/fvets.2022.888207 (PMC9354845; doi:10.3389/fvets.2022.888207)
Supplement: Supplementary file 1 [file Data_Sheet_1.ZIP › Supplementary file/miRDeep2 file.zip!/miRDeep2 file/Know_Structure/SPLP42A2/oar-mir-23a.pdf]

miRBase precursor : oar-mir-23a
Total read count : 78790
oar-miR-23a read count : 39429
oar-miR-23b read count : 39355
remaining reads : 6

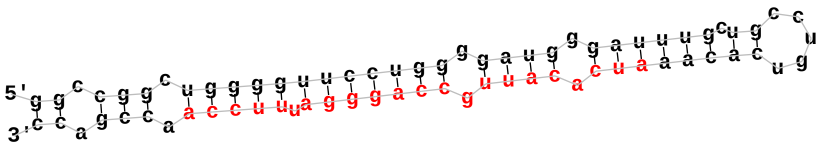

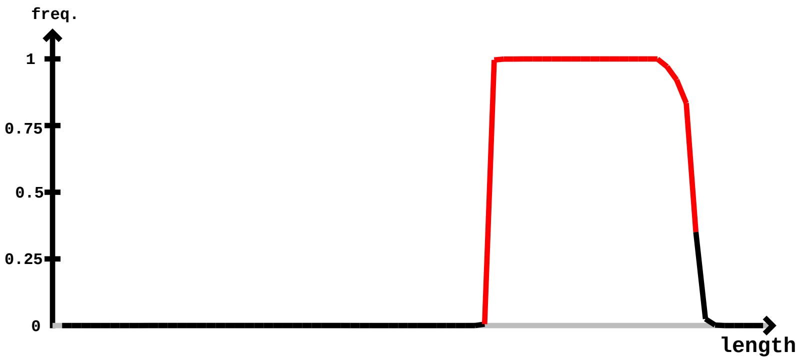


```
                                            oar-miR-23a
                                            oar-miR-23b
5'- ggccggcugggguuccuggggaugggauuugcugccugucacaaaucacauugccagggauuuccaaccgacc   -3'    exp
    ((.(((.((((((((((((.((((.((((((.((.....)))))))).)))).)))))))).))))).))).))   reads      mm         sample
    ........gggguuccuggggaugggauu............................................   4          0          seq
    ........gggguuccuggggaugggauuu...........................................   2          0          seq
    ..........................................aaaucacauugccagggauuucc........   1          0          seq
    ..........................................aaaucacauugccagggauuucca.......   1          0          seq
    ...........................................aaucacauugccagggauu...........   5          0          seq
    ...........................................aaucacauugccagggauuu..........   15         0          seq
    ...........................................aaucacauugccagggauuuc.........   19         0          seq
    ...........................................aaucacauugccagggauuucc........   130        0          seq
    ...........................................aaucacauugccagggauuucca.......   33         0          seq
    ...........................................aaucacauugccagggauuuccaa......   3          0          seq
    ............................................aucacauugccagggau............   8          0          seq
    ............................................aucacauugccagggCuu...........   3          1          seq
    ............................................aucacauugccUgggauu...........   1          1          seq
    ............................................aucacauugcUagggauu...........   3          1          seq
    ............................................aucacauugccagggauu...........   1121       0          seq
    ............................................aucacauugccagggauuu..........   1935       0          seq
    ............................................aucacauugccagggauuuc.........   3396       0          seq
    ............................................aucacauugccagggauuucc........   18875      0          seq
    ............................................aucacauugccagggauuucca.......   12770      0          seq
    ............................................aucacauugccagggauuuccaa......   896        0          seq
    ............................................aucacauugccagggauuuccaac.....   63         0          seq
    .............................................ucacauugccagggauu...........   10         0          seq
    .............................................ucacauugccagggauuu..........   6          0          seq
    .............................................ucacauugccagggauuuc.........   12         0          seq
    .............................................ucacCuugccagggauuuc.........   2          1          seq
    .............................................ucacauugccagggauuucc........   46         0          seq
    .............................................ucacauugccagggauuucca.......   36         0          seq
    .............................................ucacauugccagggauuuccaa......   7          0          seq
    .............................................ucacauugccagggauuuccaac.....   11         0          seq
    ..............................................cacauugccagggauuucca.......   1          0          seq
    ...............................................acauugccagggauuucc........   12         0          seq
    ...............................................acauugccagggauuucca.......   7          0          seq
    ...............................................acauugccagggauuuccaa......   1          0          seq
```
